# Prediction of HER2 expression in breast cancer patients based on multi-parametric MRI intratumoral and peritumoral radiomics features combined with clinical and imaging indicators

**DOI:** 10.3389/fonc.2025.1531553

**Published:** 2025-06-09

**Authors:** Xiaoxiao Li, Junfang Fang, Fuqian Wang, Lin Zhang, Xingyue Jiang, Xijin Mao

**Affiliations:** ^1^ School of Medicine, Cheeloo College of Medicine, Shandong University, Jinan, Shandong, China; ^2^ Department of Radiology, Binzhou Medical University Hospital, Binzhou, Shandong, China

**Keywords:** multi-parameter MRI, intratumoral and peritumoral, radiomics, predictive model, breast cancer, HER2

## Abstract

**Objective:**

To preoperatively evaluate the HER2 status in breast cancer using multiparametric MRI intratumoral and peritumoral radiomics features combined with clinical and imaging characteristics.

**Methods:**

This retrospective study included 252 patients with pathologically confirmed breast cancer (mean age, 50.1 ± 10.1 years) who underwent breast MRI at our hospital. Among them, 202 patients (70 HER2-positive and 132 HER2-negative) were randomly divided into a training set (n = 141) and testing set (n = 61) in a 7:3 ratio from July 2020 to December 2021. The external validation set consisted of 50 breast cancer cases (20 HER2-positive and 30 HER2-negative) from September 2024 to March 2025. Radiomics features extracted from intratumoral and peritumoral regions of the tumor on axial dynamic contrast-enhanced MRI (DCE-MRI), apparent diffusion coefficient (ADC), and T2-weighted fat-suppressed (T2FS) sequences were subjected to dimensionality reduction and model construction using Pearson correlation coefficients, recursive feature elimination, and logistic regression. Univariate and multivariate logistic regression was used to identify the independent risk factors in clinical, pathological and conventional MRI data for constructing the clinical imaging model. The combined model was built from radiomics and clinical imaging features. The area under the receiver operating characteristic curves (AUCs) were used to evaluate the predictive performance of the models.

**Results:**

There were significant statistical differences between the HER2-positive and HER2-negative groups in terms of PR expression (p=0.041), spiculation sign (p<0.001), and uneven margins (p=0.005). The AUC of radiomics models based on DCE, T2FS, and ADC sequences were 0.742, 0.748, 0.791 respectively in the training set, and 0.776, 0.708, 0.713 respectively in the testing set. The AUC of the combined clinical-radiomics model in the training set, testing set and external validation set was 0.923, 0.915 and 0.837, respectively, which was higher than the intratumoral and peritumoral radiomics model based on DCE+T2FS+ADC sequences (0.854,0.748 and 0.770) and clinical imaging model (0.820,0.789 and 0.709).

**Conclusions:**

The combined model based on DCE+T2FS+ADC intratumoral and peritumoral radiomics integrating with clinical imaging features can better predict the HER2 expression status of breast cancer.

## 1 Introduction

Breast cancer remains a major threat to women’s health, with its incidence steadily rising in recent years (1). Studies have shown that breast cancer has become the most common malignancy in females and has a trend towards younger age groups (2). Breast cancer is a highly heterogeneous tumor, with different subtypes exhibiting distinct clinical and imaging features and significant differences in prognosis. The expression status of human epidermal growth factor receptor-2 (HER2) is one of the important biological factors affecting the survival of breast cancer patients. HER2-positive breast cancer cells have strong proliferation and invasion abilities, and are prone to metastasis (3–6). Molecular targeted drugs, such as trastuzumab, can significantly improve the prognosis of HER2-positive breast cancer patients (7, 8). Therefore, accurate HER2 status assessment is crucial for prognostic evaluation and treatment strategies in breast cancer. Traditional methods primarily rely on immunohistochemistry (IHC) and fluorescence *in situ* hybridization (FISH) to evaluate HER2 receptor expression levels (9), both of which are invasive and costly. However, due to insufficient biopsy samples and tumor heterogeneity, the final detection results may fail to accurately represent the entire tumor (10).

Currently, multiparametric breast MRI is recommended as the first-line imaging modality for high-risk women with breast cancer, enabling diagnosis of malignancies, non-invasive assessment of therapeutic response, and detection of residual tumors post-surgery (11). Radiomics refers to the extraction of high-throughput, quantifiable data features from conventional medical images, highlighting subtle image characteristics imperceptible to the naked eye, thereby enabling comprehensive and precise analysis of lesions. Recent studies have shown that radiomics models based on MRI images have certain value in identifying benign and malignant lesions, distinguishing different subtypes of breast cancer, predicting axillary lymph node metastasis, and predicting the response to neoadjuvant chemotherapy and the risk of tumor recurrence, among other prognostic factors (12–15).

In recent years, radiomics based on dynamic contrast-enhanced magnetic resonance imaging (DCE-MRI) has demonstrated significant potential for the noninvasive prediction of HER2 status in breast cancer. Early studies have preliminarily demonstrated the potential of DCE-MRI texture analysis in HER2 2+ status prediction (16), while integrating semi-quantitative kinetic parameters can further improve predictive performance (17). Furthermore, Fang et al. and Xu et al. achieved a stable predictive efficacy in a multicenter cohort by constructing clinical-radiomics line graph, highlighting the clinical value of multimodal feature fusion (18, 19). However, existing research still has the following limitations: most models rely on a single DCE sequence, failing to systematically evaluate the complementary value of multi-parameter imaging such as T2-weighted fat-suppressed (T2FS) sequence and apparent diffusion coefficient (ADC); the potential impact of peritumoral microenvironment heterogeneity on HER2 status remains underexplored; the synergistic predictive efficacy between conventional imaging features, such as tumor morphology and enhancement kinetics, and radiomics features is still unclear. Therefore, the purpose of this study is to investigate the differences of intratumoral and peritumoral radiomics models based on DCE, T2FS, and ADC sequence for HER2 status assessment, and to explore the value of multi-parameter radiomics model combined with clinical imaging features in predicting HER2 expression status in breast cancer patients.

## 2 Materials and methods

### 2.1 Patients

This retrospective study was approved by the Ethics Committee of our institution, and the requirement for informed consent was waived.

**Figure 1:**
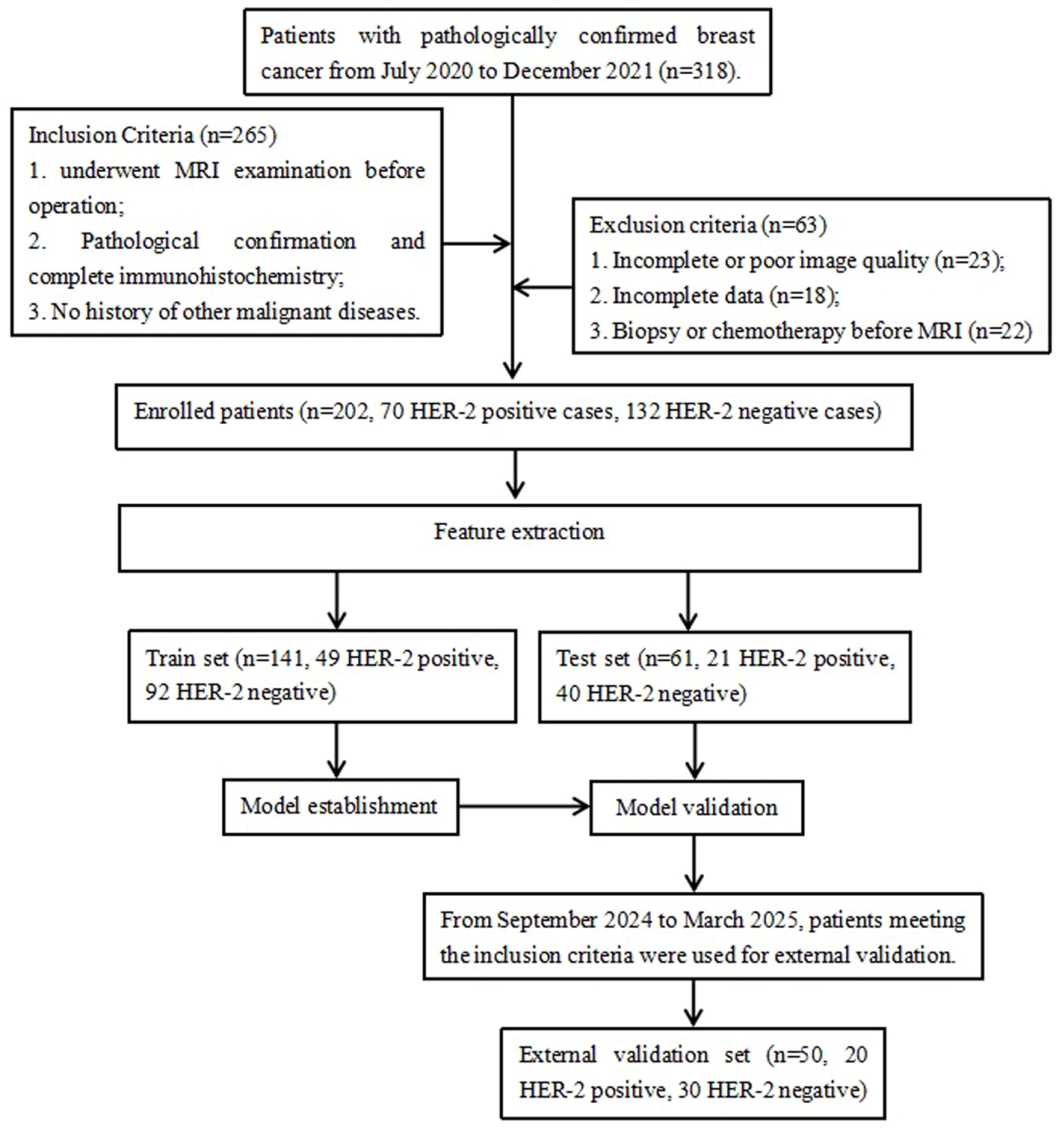
Flowchart of the recruitment pathway for patients.

A total of 318 patients with pathologically confirmed breast cancer from July 2020 to December 2021 at our hospital were retrospectively collected in this study (**Figure 1**). The inclusion criteria were: (i) Underwent preoperative MRI examination with Diffusion-Weighted Imaging (DWI), T2FS and DCE sequences; (ii) Pathological confirmed breast cancer and completed immunohistochemistry; (iii) No history of other malignant diseases. The exclusion criteria were: (i) Incomplete or poor quality MR images, and non-mass enhancement lesions difficult to delineate; (ii) incomplete clinical and/or pathology data; (iii) preoperative radiotherapy, chemotherapy, or biopsy. Finally, 202 patients with breast cancer were enrolled, including 70 HER2-positive cases and 132 HER2-negative cases. All patients were female, with an age ranged from 23 to 79 years and a mean age of 52.1 ± 10.3 years. The patients’ age, maximum tumor cross-sectional area, estrogen receptor (ER) status, progesterone receptor (PR) status, Ki-67 status, histological grade, and lymph node metastasis data were collected using the hospital HIS and PACS systems. The 50 cases of breast cancer (20 HER2-positive and 30 HER2-negative) from September 2024 to March 2025 were used as the external validation set, all female, with an age ranged from 22 to 83 years and a mean age of 48.2 ± 10.3 years.

### 2.2 Imaging acquisition

All breast MRI examinations were performed with the patient in the prone position using a 3.0T MRI scanner from Siemens AG, Germany, equipped with a 4-channel dedicated breast coil. The scanning range extended from the axilla to the lower margin of the breasts. T1-weighted images (TR169ms/TE2.6ms), T2-weighted images (3500ms/69ms), fat-suppressed T2-weighted images (3200ms/69ms), and diffusion-weighted imaging (DWI) (6500ms/65ms) with a b value of 800s/mm2 were acquired. The axial slice thickness was 4mm with a 0.4mm gap, and the field of view (FOV) was 340mm × 340mm. For the DCE-MRI, a total of 7 to 8 phases (1 phase before enhancement and 6 or 7 phases after enhancement) were obtained using VIBE sequence (FOV 340 mm × 340 mm, TR 4.49 ms, TE 1.68 ms, thickness 1.2 mm, space 0.24 mm, matrix 352 × 260, flip angle 10°). At the end of the first scan, Gd-DTPA (Germany, Berlin, Schering Co., Ltd. (SCHERING)) was injected at a dose of 0.2 ml/kg via antecubital vein using pressure injector at the rate of 2.5 ml/s, which was followed by 20 ml saline flush.

The apparent diffusion coefficient (ADC) map and the time-signal intensity curve (TIC) were obtained on the syngoMMWP VE40B workstation. The MRI imaging information of the lesions were evaluated according to the American College of Radiology’s Breast Imaging Reporting and Data System (BI-RADS) 2023 edition.

### 2.3 Radiomics model establishment

#### 2.3.1 Image segmentation and radiomics feature extraction

**Figure 2:**
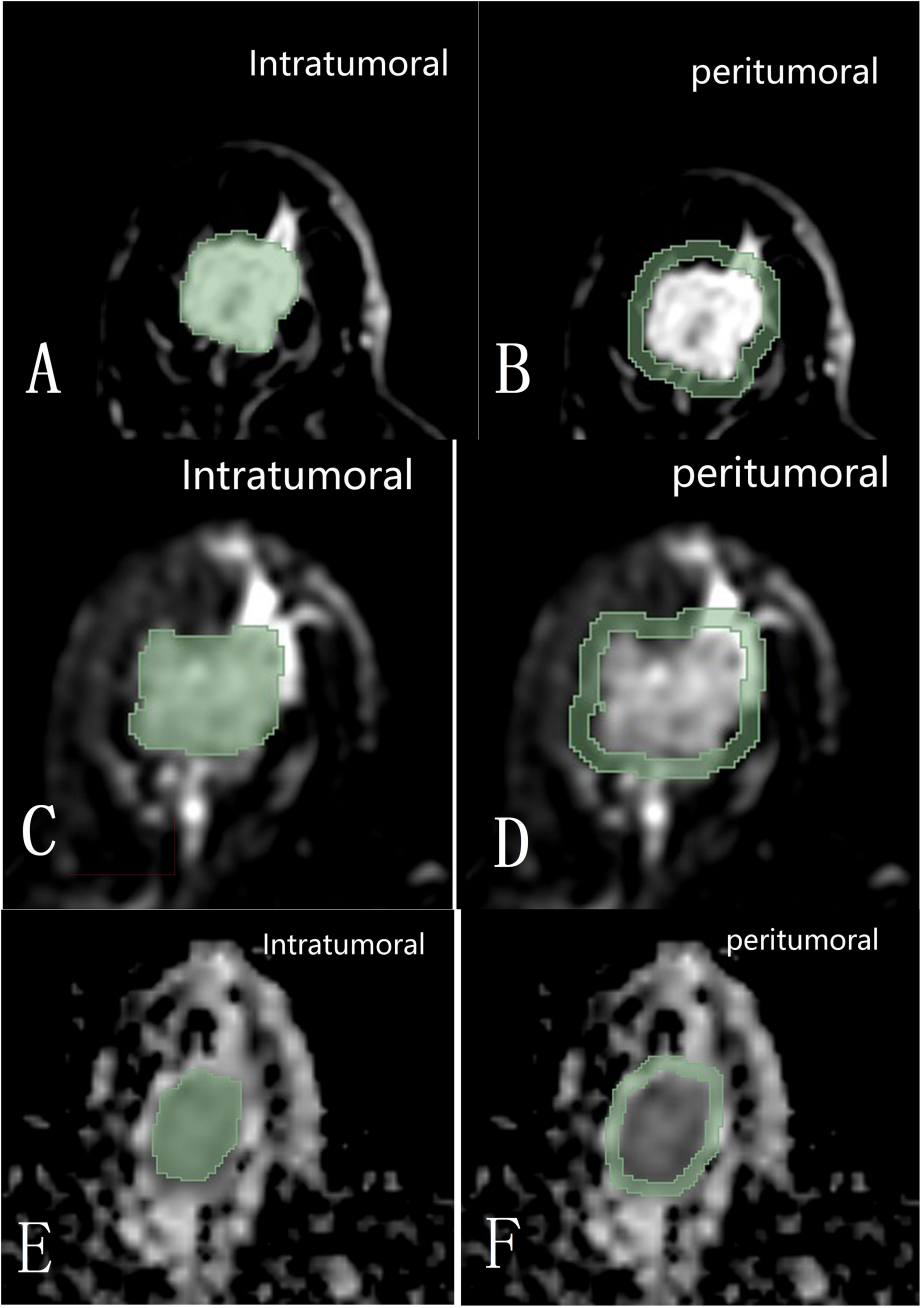
Diagram shows the intratumoral and 3 mm peritumoral image regions within the maximum cross-section on DCE **(A, B)**, T2FS **(C, D)**, and ADC **(E, F)** sequences of the tumor delineated with 3D Slicer 4.11 software.

The 3D Slicer 4.11 software was used to delineate the ROIs within and around the tumors at its maximum cross-section on axial T2FS, ADC, and DCE sequences. The intratumoral region did not exceed the tumor boundary, and the peritumoral region was selected to be 3 millimeter beyond the tumor boundary (**Figure 2**). The delineation of DCE sequence select the fourth image, T2FS and ADC map delineation level need to match with the enhancement sequence. The ROIs were manually drawn by two radiologists (Reader 1, with 6 years of experience in breast image interpretation, and Reader 2, with 18 years of experience), who were blinded to the clinical and histopathological data of the patients. Forty cases’ delineation were randomly selected to test the consistency between Reader 1 and 2.

Radiomics feature extraction and analysis were performed using the SlicerRadiomics plugin in 3D Slicer 4.11 software. The radiomics features our study extracted include statistical features, texture features and high-order features. Before texture feature extraction, wavelet and Laplacian of Gaussian (LoG) were used for pre-processing. A total of 1023 radiomics features, namely, 14 shape features, 162 first-order histogram features, and 847 texture features were extracted.

#### 2.3.2 Feature selection and model construction

**Figure 3:**
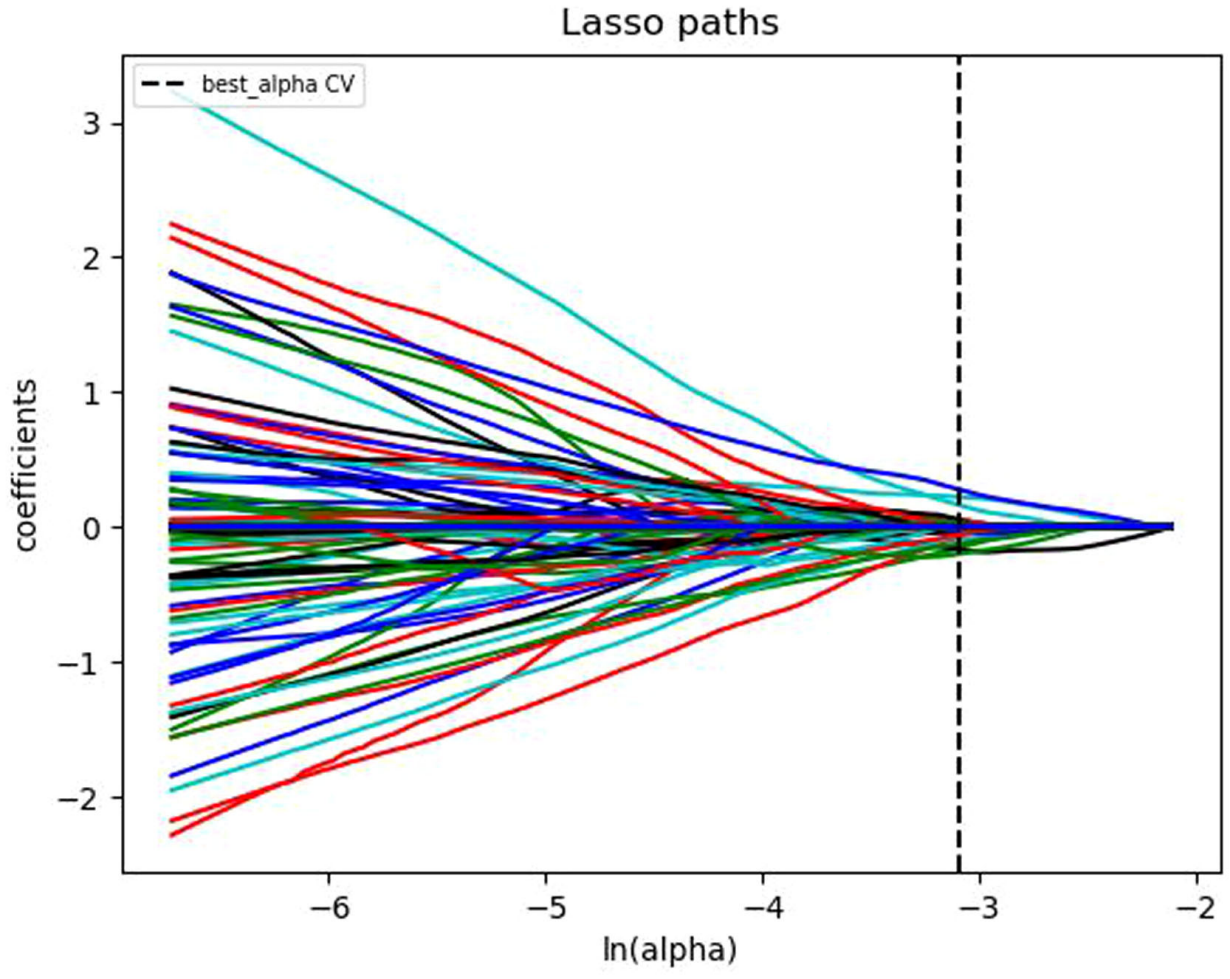
Convergence plot of feature coefficients selected by LASSO algorithm based on T1-weighted DCE-MRI for optimal radiomics features.

The extracted single and multi-modal radiomics features were normalized using the Z-score normalization method. The data was divided into a training set and a testing set in a 7:3 ratio. For the training set, the variance threshold method and the least absolute shrinkage and selection operator (LASSO) algorithm (**Figure 3**) were used to reduce dimensionality, eliminate redundant eigenvalues, select radiomics features, and calculate the Rad score.

The radiomics-based analysis was performed to calculate the radiomics signature (RAD) values for the three sequences. The formula for RAD value calculation is as follows (✱ represents peritumoral features):


$$\begin{array}{l} \text{DCE}\;\text{RAD}\;\text{Score}=-0.7629+(-0.7713\times \text{Kurtosis}.3)+(–0.3904\times \text{Mean}.5)\\ +(-0.0062\times \text{Median}.5)\;+(-0.6680\times \text{ClusterShade}*)\\ +(-0.0771\times \text{SizeZoneNonUniformityNormalized}.5*)\\ +0.5381\times \text{Idn}.1*+0.6774\times \text{Kurtosis}.9*+0.5109\times \text{MCC}.5 \end{array}$$

$$\begin{array}{l} \text{T}2\text{FS}\;\text{RAD}\;\text{Score}=-0.8076\;+\;5.0559\times \text{SmallAreaLowGrayLevelEmphasis}.1\\ +\;(-5.6557\times \text{Contrast}.5)\\ +\;(-0.4640\times \text{Kurtosis}.3*)\\ +\;(-0.0695\times \text{Mean}.3*)+0.2525\times \text{Median}.5*\\ +\;\;0.4529\times \text{Skewness}.9*+(-0.6584\times \text{Skewness}.10*) \end{array}$$

$$\begin{array}{l} \text{ADC}\;\text{RAD}\;\text{Score}=-0.9303+\;(-1.4610\times \text{Coarseness}.9)+(-0.28\times \text{LargeDependenceEmphasis}.6\;)\\ +\;(-0.3157\times \text{Idn}.1)+(-1.5059\times \text{ClusterShade}.4\;)\\ +\;0.8592\times \text{Imc}2.8*+0.0477\times \text{Skewness}.8*\\ +0.1415\times \text{Skewness}.5* \end{array}$$

The statistical information of clinical, pathological, imaging and radiomics features were included in the logistic regression algorithm, and 10-fold cross-validation was used to construct the prediction model. The area under the receiver operating characteristic (ROC) curves (AUCs) were used to evaluate the predictive performance of the models.

#### 2.3.3 Pathological assessment

HER2 detection was performed using immunohistochemistry (IHC), and the HER2 expression level was recorded as the percentage of malignant cells stained positive by two experienced pathologists. HER2 IHC Scoring Criteria: IHC 0: No staining or ≤10% of invasive cancer cells with incomplete/faint membrane staining. IHC 1+: >10% of invasive cancer cells with incomplete/faint membrane staining. IHC 2+: >10% of invasive cancer cells with weak to moderate complete membrane staining, or ≤10% of invasive cancer cells with strong complete membrane staining. IHC 3+: >10% of invasive cancer cells with strong, complete, and uniform circumferential membrane staining (20, 21). The IHC result of 3+ was considered HER2-positive, while the IHC result of 0 or 1+ was considered HER2-negative. For tumors with the IHC result of 2+, fluorescence *in situ* hybridization (FISH) analysis was required. Those with amplification were HER2-positive, while those without amplification were HER2-negative (20, 21).

#### 2.3.4 Statistical analysis

The clinical data were analyzed by SPSS 25.0 software. Independent sample t-tests were used for inter-group comparisons when the data met the normal distribution, otherwise the Mann-Whitney U test was used. Univariate and multivariate logistic regression were used to select clinical, pathological, and imaging features. R 3.4.4 software was performed for modeling and drawing ROC curves and nomograms to evaluate the value of each feature. The DeLong test was used to compare the predictive models, with p<0.05 indicating a significant difference. The intraclass correlation coefficient (ICC) analysis was used to evaluate the reproducibility and stability of radiomics feature extraction, and ICC≥0.75 indicated good consistency.

## 3 Results

### 3.1 Patient characteristics

The clinicopathological features and imaging characteristics of the HER2-positive group (n = 70) and HER2-negative group (n = 132) were listed in **Table 1**. Univariate logistic regression analysis showed that there were statistically significant differences between the two groups in ER expression (p<0.001), PR expression (p<0.001), spiculation sign (p<0.001), uneven margins (p=0.019), and TIC type (p=0.007). Multivariate logistic regression was performed on the characteristics with statistical differences (**Table 2**), the expression of PR (p=0.041), spiculation sign (p<0.001) and uneven margins (p=0.005) were significantly different between HER2-positive group and HER2-negative group, which were included in the construction of the clinical imaging model.

**Table 1:** Univariate logistic regression analysis of clinicopathological data and image characteristics of breast cancer patients in HER2-positive group and HER2-negative group.

Parameters		HER2 expression status		B Value	Wald	P Value	OR (95%CI)
		Positive (n=70)	Negative (n=132)				
Age (years)		49.810 ± 9.990	50.400 ± 10.210	-0.006	0.155	0.694	0.994 (0.966-1.023)
Maximum cross-sectional area		4.400 (2.975 , 7.325)	3.500 (2.000, 6.800)	0.011	0.589	0.443	1.011 (0.983-1.04)
ADC value		917.700 ± 178.590	884.630 ± 189.170	0.001	1.443	0.230	1.001 (0.999-1.003)
Ki-67		40.0 (27.5 , 50.0)	30.0 (15.0, 50.0)	0.013	3.763	0.052	1.013 (1.000-1.027)
ER	0	34	32	-0.452	16.172	p<0.001	0.636 (0.510-0.0.793)
	1+	4	3				
	2+	10	16				
	3+	22	81				
PR	0	44	43	-0.616	19.318	p<0.001	0.54 (0.410-0.711)
	1+	11	18				
	2+	8	31				
	3+	7	40				
Histological grade	I	4	9	-0.034	0.199	0.655	0.967 (0.833-1.122)
	II	44	92				
	III	22	30				
Spiculation sign	No	49	37	-1.790	30.356	p<0.001	0.167 (0.088-0.316)
	Yes	21	95				
Necrosis	No	28	68	0.466	2.418	0.120	1.594 (0.886-2.868)
	Yes	42	64				
Uneven margins	No	37	47	-0.707	5.530	0.019	0.493 (0.274-0.889)
	Yes	33	85				
TIC	I	0	2	0.855	7.236	0.007	2.351 (1.261-4.383)
	II	18	57				
	III	52	73				
Lymph Node Metastasis	No	43	47	-0.314	1.083	0.298	0.731 (0.405-1.319)
	Yes	27	85				

ADC, Apparent diffusion coefficient.

Ki-67, Ki-67 Antigen.

ER, Estrogen Receptor.

PR, Progesterone Receptor.

TIC, Time-signal intensity curve.

**Table 2:** Multivariate logistic regression analysis of clinicopathological and imaging features between HER2-positive group and HER2-negative group.

Parameters	B value	Wald	P value	OR (95%CI)
ER	-0.170	0.908	0.341	0.844 (0.595-1.197)
PR	-0.440	4.178	0.041	0.644 (0.422-0.982)
Spiculation sign	-1.574	16.562	0.000	0.207 (0.097-0.442)
Uneven margins	-1.017	7.816	0.005	0.362 (0.177-0.738)
TIC	0.492	1.447	0.229	1.635 (0.734-3.645)

ER, Estrogen Receptor.

PR, Progesterone Receptor.

TIC, Time-signal intensity curve.

### 3.2 ICC analysis

For the forty cases’ delineation with the same lesion, reader 1 and reader 2 outlined the ROI of the multi-parametric images and the ICC values of the obtained imaging features were all greater than 0.75, indicating good consistency.

### 3.3 Radiomics feature extraction

The 202 patients with breast cancer were randomly divided into a training set of 141 cases and a testing set of 61 cases in a 7:3 ratio. After radiomics feature extraction and dimensionality reduction, the optimal features for the DCE sequence included 4 intratumoral and 4 peritumoral features, for the T2FS sequence included 2 intratumoral and 5 peritumoral features, and for the ADC sequence included 4 intratumoral and 3 peritumoral features.

### 3.4 Construction of radiomics model

**Figure 4:**
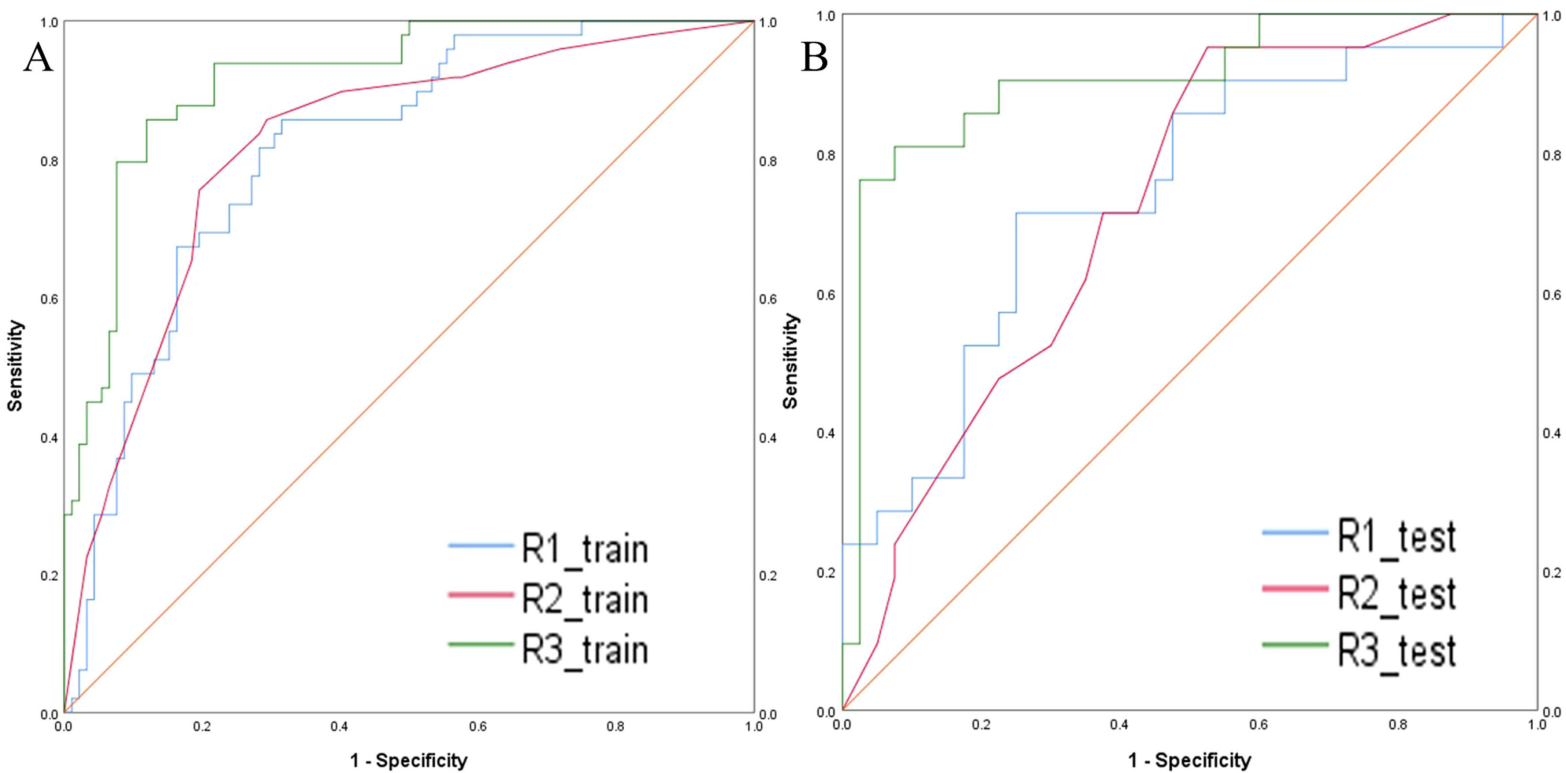
ROC curves of the models build from radiomic features of DCE, T2FS, and ADC in the training set **(A)** and testing set **(B)** for predicting the HER2 status of breast cancer.

The intratumoral and peritumoral features from DCE, T2FS and ADC sequence were included into multi-factor logistics regression analysis to construct the radiomics models. The predictive performance of the models built from radiomics features from DCE alone, T2FS alone, and ADC alone were summarized in **Table 3**. The AUC values of the testing set from the DCE, T2FS, and ADC sequences were 0.776 (95% CI 0.662-0.879), 0.708 (95% CI 0.589-0.821), and 0.713 (95% CI 0.6-0.823), respectively (**Figure 4**).

**Table 3:** Predictive performance of the models build from radiomics features from DCE alone, T2FS alone, and ADC alone.

Models		ROC	ROC confidence interval	Sensitivity	Specificity
DCE	Training set	0.742	0.670-0.812	0.429	0.935
	Testing set	0.776	0.662-0.879	0.531	0.793
T2FS	Training set	0.748	0.676-0.815	0.653	0.783
	Testing set	0.708	0.589-0.821	0.286	0.891
ADC	Training set	0.791	0.722-0.856	0.776	0.772
	Testing set	0.713	0.600- 0.823	0.408	0.891

DCE, dynamic contrast-enhanced T1-weighted fat-saturated sequence.

T2FS, fat-suppressed T2-weighted.

ADC, apparent diffusion coefficient.

### 3.5 Construction of combined model

**Figure 5:**
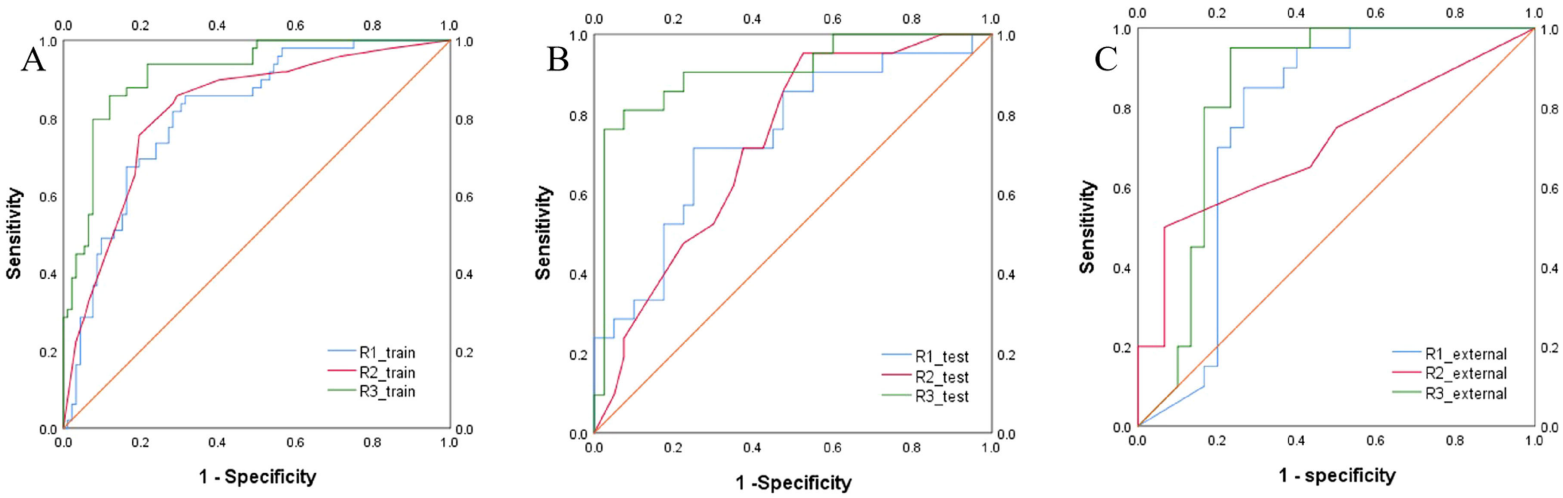
ROC curves of the R1, R2, and R3 models in the training set **(A)**, testing set **(B)** and external validation set **(C)**) for predicting the HER2 status of breast cancer.

**Figure 6:**
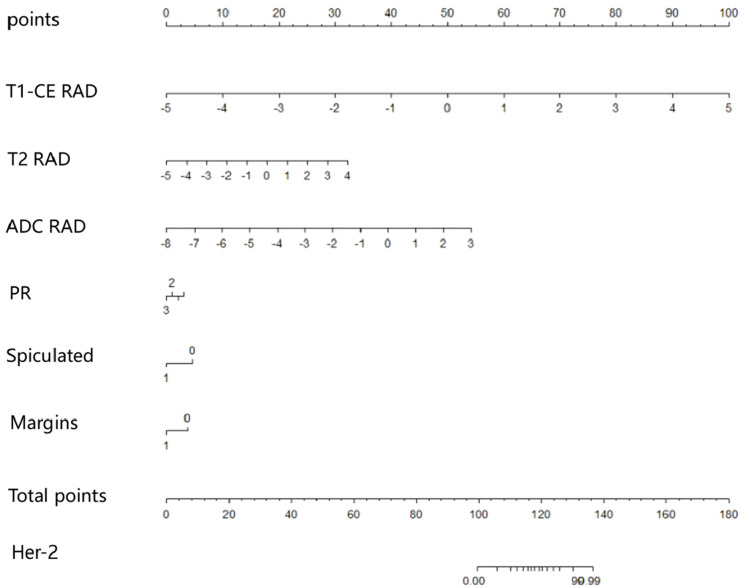
Nomogram of the six variables in the combined prediction model.

**Figure 7:**
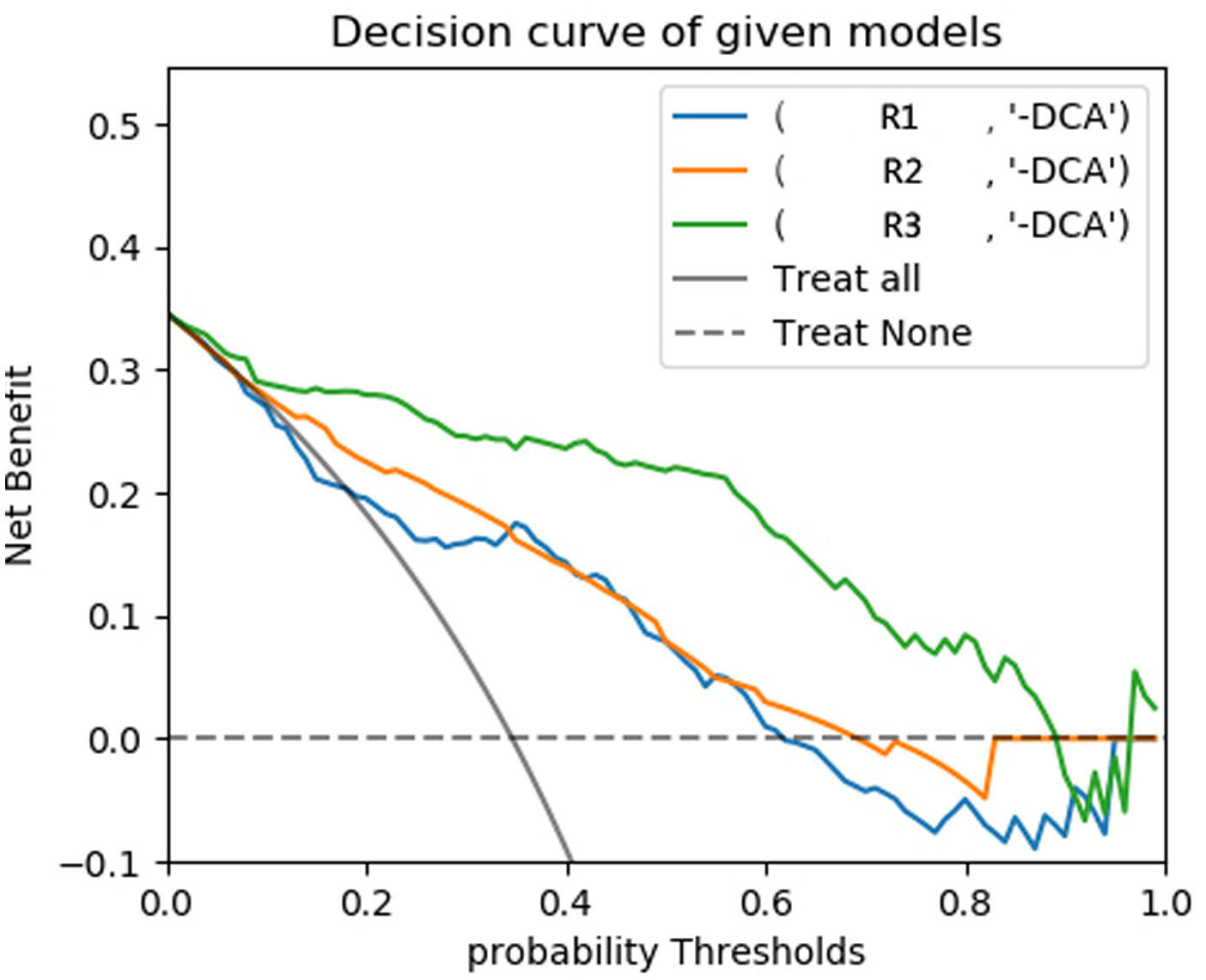
DCA curves of testing set for each model.

The predictive models were constructed using logistic regression algorithms based on the statistically significant of intratumoral and peritumoral radiomics features from DCE+T2FS+ADC sequence (R1), clinicopathological imaging features including PR expression status, spiculation sign, and uneven margins (R2), and a combination of both (R3). The diagnostic efficacies of the three models were summarized in **Table 4**. The AUC values of the combined model (R3) in the training set, the testing set and external validation set was 0.923, 0.915 and 0.837, respectively, which was superior to the R1 model and R2 model (**Figure 5**). DeLong test showed that there was a significant difference between model R3 and other individual models (**Table 5**). The nomogram of the six variables in the combined prediction model was plotted (**Figure 6**), and the clinical practicality of R3 been validated through decision curve analysis (**Figure 7**).

**Table 4:** Diagnosis efficacy of the R1, the R2, and the R3 model.

Models		ROC	ROC confidence interval	Sensitivity	Specificity
R1	Training set	0.854	0.801-0.905	0.816	0.826
	Testing set	0.748	0.669-0.883	0.490	0.880
	External validation set	0.770	0.632-0.909	0.730	0.780
R2	Training set	0.820	0.757-0.880	0.857	0.707
	Testing set	0.789	0.607-0.824	0.918	0.435
	External validation set	0.709	0.557-0.861	0.930	0.760
R3	Training set	0.923	0.886-0.959	0.891	0.902
	Testing set	0.915	0.865- 0.985	0.989	0.902
	External validation set	0.837	0.717-0.956	0.770	0.840

**Table 5:** DeLong test analysis for the R1, R2, and R3 models.

Models	P value		
	R1	R2	R3
R1	1	>0.05	<0.001
R2	>0.05	1	<0.001
R3	<0.001	<0.001	1

## 4 Discussion

HER2 is a proto-oncogene that plays a critical role in regulating cell growth, differentiation, and metastasis (2). Predicting the expression status of HER2 in breast cancer patients non-invasively and accurately before surgery is of considerable clinical significance. This study constructed models based on DCE, T2FS, and ADC intratumoral and peritumoral radiomics features and clinical imaging characteristics to predict the expression status of HER2 in breast cancer patients. It innovatively proposed that the expression status of PR, the spiculation sign and uneven margins in conventional imaging have predictive value for HER2 expression in breast cancer patients. The results demonstrated that the combined model, which integrates multi-sequence intratumoral and peritumoral radiomics features with PR receptor expression, spiculation sign, and uneven margins, showed good predictive performance for HER2 expression status in both the training set, testing set and external validation set. The construction of the nomogram provides a simple and intuitive method for predicting HER2 expression in clinical practice.

This study used DCE, T2FS, and ADC sequences for image segmentation. For the DCE-MRI images, we selected the fourth phase, which is 180 seconds after the intravenous injection of the contrast agent. This time point belongs to the beginning of the delayed period, and the enhancement degree of different TIC types of breast cancer is relatively obvious, which not only reflects the tumor’s blood perfusion characteristics, but also maintains a high contrast between the tumor and surrounding normal tissue (22, 23). This ensures that the extracted feature data is more representative and distinguishable. Additionally, previous studies have primarily focused on features within the intratumoral region (24), while peritumoral features also contain key information about breast cancer (25, 26). Therefore, this study extracted radiomics features from both intratumoral region and peritumoral region, and constructed a model based on the optimal feature subsets of the corresponding regions. Logistic regression was used to construct models based on the DCE, T2FS, and ADC sequences, respectively, to evaluate the HER2 expression status of breast cancer in the testing set. The results showed that the radiomics features extracted from the DCE sequence had better evaluation efficacy than those extracted from the T2FS or ADC sequence. This could be due to the fact that HER2 gene amplification is related to accelerated tumor neovascularization and invasiveness (5). And compared with the T2FS and ADC sequences, the DCE sequence can better reflect tumor blood flow, vascular density, and vascular permeability (27). In addition, previous study reported that the AUC value of radiomics model build from the DCE sequence to predict the HER2 expression status was only 0.65 (28), while the AUC value in this study was 0.776. This may be because this study simultaneously selected four intratumoral and four peritumoral features from the DCE sequence, which could more comprehensively reflect the characteristics of the tumor and the tumor’s surrounding microenvironment.

Studies have shown that there might be some correlation between HER2 status and certain features of conventional imaging methods, such as spiculated margins, heterogeneous enhancement, and microcalcifications, but the performance of these features in predicting the HER2 status is limited and the results are still controversial (29). Through univariate and multivariate logistic regression analyses on clinical, pathological, and imaging features, our study found that PR expression, spiculation sign, and uneven margins were independent predictors of HER2 expression status. Arpino et al. pointed out that PR loss may be a surrogate marker for excessive growth factor receptor activation. In our research, PR-negative status was significantly associated with HER2-positive expression, which may be related to the poor prognosis of HER2-positive breast cancer (30). Sturesdotter et al. proposed that the spiculation sign of breast tumors represents a desmoplastic response in the adjacent stroma or periductal fibrosis, which is associated with lower histologic grade and lower Ki-67 values, indicating a favorable biological behavior of the tumor (29). This study is consistent with previous research that speculation sign was more commonly observed in the HER2-negative group with better prognosis compared to HER2-positive breast cancer. Previous studies have indicated that HER2-positive breast cancer often presents with indistinct margins on ultrasound (31). However, the results of this study showed the uneven margins were more common in the HER2-negative group. The authors speculate that HER2-positive breast cancer tends to grow faster and larger than HER2-negative breast cancer, and is more likely to compress surrounding tissues, which makes the tumor margins clearer. The inconsistency with previous literature may be attributed to differences in sample sizes between studies and the heterogeneity of tumors. Additionally, variations in imaging techniques could also contribute to these discrepancies. In terms of margin assessment, ultrasound primarily evaluates the sharpness and regularity of tumor margins through two-dimensional grayscale images, which can be influenced by the operator’s skill and equipment quality, leading to subjective differences among physicians. In contrast, the high resolution of MRI can more clearly show the three-dimensional structure of lesion margins.

The combined model of this study based on multi-parametric MRI radiomics and clinical imaging features showed better performance in evaluating HER2 expression status than the individual model, with higher evaluation efficiency and good prediction ability in both the training set, testing set and external validation set. A statistically significant difference between models R1 and R3, as well as R2 and R3, was shown by the DeLong test. These results imply that the clinicopathology, imaging and radiomics information complement each other and can more comprehensively represent tumor features, exhibiting strong predictive performance in assessing HER2 status of breast cancer. However, our study has some limitations. First, it is a retrospective study, which may have some selection bias. Second, as a single-center study, the sample size is relatively small, and multi-center participation and large-scale prospective studies are needed to further validate the efficacy of nomogram. Third, in this study, radiomics analysis was only performed on two-dimensional images of the maximum cross-sectional area of the tumor, which may miss some important information as compared to a model based on features of the whole tumor volume.

In conclusion, this study suggests that the combined model based on multi-parametric MR intratumoral and peritumoral radiomics combined with clinical imaging features can better predict the HER2 expression status of breast cancer. It is expected to become a reliable method for evaluating HER2 status in breast cancer patients.

## Data Availability

The original contributions presented in the study are included in the article/supplementary material. Further inquiries can be directed to the corresponding author.

## References

[1] Siegel RL Miller KD Fuchs HE Jemal A. Cancer statistics, 2022. CA Cancer J Clin. (2022) 72:7–33. doi: 10.3322/caac.21708 35020204

[2] Sung H Ferlay J Siegel RL Laversanne M Soerjomataram I Jemal A. Global Cancer Statistics 2020: GLOBOCAN Estimates of Incidence and Mortality Worldwide for 36 Cancers in 185 Countries. CA Cancer J Clin. (2021) 71:209–49. doi: 10.3322/caac.21660 33538338

[3] Elshazly AM Gewirtz DA. An overview of resistance to Human epidermal growth factor receptor 2 (Her2) targeted therapies in breast cancer. Cancer Drug Resist. (2022) 5:472–86. doi: 10.20517/cdr.2022.09 PMC9255238 35800378

[4] Zhang H Ren G Wang X Zhao J Yao H Bai Y. HER2 gene amplification by fluorescence in situ hybridization (FISH) compared with immunohistochemistry (IHC) in breast cancer: a study of 528 equivocal cases. Breast Cancer Res Treat. (2012) 134:743–9. doi: 10.1007/s10549-012-2101-x 22678158

[5] Kang SS Ko EY Han B-K Shin JH Hahn SY Ko ES. Background parenchymal enhancement on breast MRI: Influence of menstrual cycle and breast composition. Magn Reson Imag. (2014) 39:526–34. doi: 10.1002/jmri.24185 23633296

[6] Cortazar P Zhang L Untch M Mehta K Costantino JP Wolmark N. Pathological complete response and long-term clinical benefit in breast cancer: the CTNeoBC pooled analysis. Lancet. (2014) 384:164–72. doi: 10.1016/S0140-6736(13)62422-8 24529560

[7] Yang L Li Y Bhattacharya A Zhang Y. A recombinant human protein targeting HER2 overcomes drug resistance in HER2-positive breast cancer. Sci Transl Med. (2019) 11:576–82. doi: 10.1126/scitranslmed.aav1620 PMC6409101 30674653

[8] Waks AG Winer EP. Breast Cancer Treatment: A Review. JAMA. (2019) 321:288–300. doi: 10.1001/jama.2018.19323 30667505

[9] Bitencourt AGV Gibbs P Rossi Saccarelli C Daimiel I Lo Gullo R Fox MJ. MRI-based machine learning radiomics can predict HER2 expression level and pathologic response after neoadjuvant therapy in HER2 overexpressing breast cancer. EBioMedicine. (2020) 61:103042. doi: 10.1016/j.ebiom.2020.103042 33039708 PMC7648120

[10] Gucalp A Traina TA. Targeting the androgen receptor in triple-negative breast cancer. Curr Probl Cancer. (2016) 40:141–50. doi: 10.1016/j.currproblcancer.2016.09.004 PMC5580391 27816190

[11] Wekking D Porcu M De Silva P Saba L Scartozzi M Solinas C. Breast MRI: Clinical Indications, Recommendations, and Future Applications in Breast Cancer Diagnosis. Curr Oncol Rep. (2023) 25:257–67. doi: 10.1007/s11912-023-01372-x 36749493

[12] Parekh VS Jacobs MA. Integrated radiomic framework for breast cancer and tumor biology using advanced machine learning and multiparametric MRI. NPJ Breast Cancer. (2017) 3:43. doi: 10.1038/s41523-017-0045-3 29152563 PMC5686135

[13] Huang Y Wei L Hu Y Shao N Lin Y He S. Multi-Parametric MRI-Based Radiomics Models for Predicting Molecular Subtype and Androgen Receptor Expression in Breast Cancer. Front Oncol. (2021) 11:706733. doi: 10.3389/fonc.2021.706733 34490107 PMC8416497

[14] Calabrese A Santucci D Landi R Beomonte Zobel B Faiella E de Felice C. Radiomics MRI for lymph node status prediction in breast cancer patients: the state of art. J Cancer Res Clin Oncol. (2021) 147:1587–97. doi: 10.1007/s00432-021-03606-6 PMC11801989 33758997

[15] Wang X Xie T Luo J Zhou Z Yu X Guo X. Radiomics predicts the prognosis of patients with locally advanced breast cancer by reflecting the heterogeneity of tumor cells and the tumor microenvironment. Breast Cancer Res. (2022) 24:20. doi: 10.1186/s13058-022-01516-0 35292076 PMC8922933

[16] Jiang Z Song L Lu H Yin J. The Potential Use of DCE-MRI Texture Analysis to Predict HER2 2+ Status. Front Oncol. (2019) 9:242. doi: 10.3389/fonc.2019.00242 31032222 PMC6473324

[17] Song L Li C Yin J. Texture Analysis Using Semiquantitative Kinetic Parameter Maps from DCE-MRI: Preoperative Prediction of HER2 Status in Breast Cancer. Front Oncol. (2021) 11:675160. doi: 10.3389/fonc.2021.675160 34168994 PMC8217832

[18] Fang C Zhang J Li J Shang H Li K Jiao T. Clinical-radiomics nomogram for identifying HER2 status in patients with breast cancer: A multicenter study. Front Oncol. (2022) 12:922185. doi: 10.3389/fonc.2022.922185 36158700 PMC9490879

[19] Xu A Chu X Zhang S Zheng J Shi D Lv S. Development and validation of a clinicoradiomic nomogram to assess the HER2 status of patients with invasive ductal carcinoma. BMC Cancer. (2022) 22:872. doi: 10.1186/s12885-022-09967-6 35945526 PMC9364617

[20] Wolff AC Somerfield MR Dowsett M Hammond MEH Hayes DF McShane LM. Human Epidermal Growth Factor Receptor 2 Testing in Breast Cancer: ASCO-College of American Pathologists Guideline Update. J Clin Oncol. (2023) 41:3867–72. doi: 10.1200/JCO.22.02864 37284804

[21] Liu YP Yang WT Liang ZY Bu H. HER2 detection guidelines for breast cancer (2024 edition). Chin J Pathol. (2024) 53:1192–201. doi: 10.3760/cma.j.cn112151-20241009-00664

[22] Wang FQ Wang S Mao XJ Di Ng Xm Xue Ly Zhang J. Study on the prediction of K-i 6–7 expression of breast carcinoma using intratumoral and peritumoral radiomics based on mult-i modal MRI by machine learning. J Pract Radiol. (2023) 39:1606–10. doi: 10.3969/j.issn.1002-1671.2023.10.010

[23] Peng S Chen L Tao J Liu J Zhu W Liu H. Radiomics Analysis of Multi-Phase DCE-MRI in Predicting Tumor Response to Neoadjuvant Therapy in Breast Cancer. Diagn (Basel). (2021) 11:2086. doi: 10.3390/diagnostics11112086 PMC8625316 34829433

[24] Zhou J Tan H Li W Liu Z Wu Y Bai Y. Radiomics Signatures Based on Multiparametric MRI for the Preoperative Prediction of the HER2 Status of Patients with Breast Cancer - ScienceDirect. Acad Radiol. (2021) 28:1352–60. doi: 10.1016/j.acra.2020.05.040 32709582

[25] Li C Song L Yin J. Intratumoral and Peritumoral Radiomics Based on Functional Parametric Maps from Breast DCE-MRI for Prediction of HER2 and Ki-67 Status. J Magn Reson Imag. (2021) 54:703–14. doi: 10.1002/jmri.27651 33955619

[26] Braman NM Etesami M Prasanna P Dubchuk C Gilmore H Tiwari P. Intratumoral and peritumoral radiomics for the pretreatment prediction of pathological complete response to neoadjuvant chemotherapy based on breast DCE-MRI. Breast Cancer Res. (2017) 19:57. doi: 10.1186/s13058-017-0846-1 28521821 PMC5437672

[27] Padhani AR Khan AA. Diffusion-weighted (DW) and dynamic contrast-enhanced (DCE) magnetic resonance imaging (MRI) for monitoring anticancer therapy. Target Oncol. (2010) 5:39–52. doi: 10.1007/s11523-010-0135-8 20383784

[28] Li H Zhu Y Burnside ES Huang E Drukker K Hoadley KA. Quantitative MRI radiomics in the prediction of molecular classifications of breast cancer subtypes in the TCGA/TCIA data set. NPJ Breast Cancer. (2016) 2:16012. doi: 10.1038/npjbcancer.2016.12 27853751 PMC5108580

[29] Sturesdotter Li Sandsveden M Johnson K Larsson A-M Zackrisson S Sartor H. Mammographic tumour appearance is related to clinicopathological factors and surrogate molecular breast cancer subtype. Sci Rep. (2020) 10:20814. doi: 10.1038/s41598-020-77053-7 33257731 PMC7705680

[30] Arpino G Weiss H Lee AV Schiff R De Placido S Osborne CK. Estrogen Receptor-Positive, Progesterone Receptor-Negative Breast Cancer: Association with Growth Factor Receptor Expression and Tamoxifen Resistance. Breast Dis. (2006) 17:1254. doi: 10.1093/jnci/dji249 16145046

[31] Mansour S Abdullah E Mohamed EH Gomaa M. Imaging of HER2 detected receptor expression positive breast cancer: from detection to interpretation. Egypt J Radiol Nucl Med. (2023) 54:122. doi: 10.1186/s43055-023-01063-4

